# Emergency Double Valve Replacement for Ruptured Mitral Valve Aneurysm and Annular Abscess Caused by Gemella Morbillorum Endocarditis in a Patient with Bicuspid Aortic Valve: A Case Report

**DOI:** 10.70352/scrj.cr.25-0659

**Published:** 2026-04-23

**Authors:** Yoshihiko Onishi, Takayuki Abe, Yuki Hirai, Taichi Kondo, Kouan Orii

**Affiliations:** Department of Cardiac Surgery, Saitama Cardiovascular and Respiratory Disease Center, Kumagaya, Saitama, Japan

**Keywords:** mitral valve aneurysm, infective endocarditis, *Gemella morbillorum*, bicuspid aortic valve

## Abstract

**INTRODUCTION:**

Mitral valve aneurysm (MVA) is a rare but potentially fatal complication of infective endocarditis (IE), often associated with aortic valve involvement. *Gemella morbillorum*, a commensal oral bacterium, is an uncommon cause of IE. We report a rare case of MVA rupture with an aortic annular abscess caused by *G. morbillorum* IE in a patient with bicuspid aortic valve (BAV), successfully managed with emergency valve replacement.

**CASE PRESENTATION:**

A 36-year-old man presented with congestive heart failure and respiratory failure. Echocardiography revealed a ruptured MVA, severe mitral and aortic regurgitation, and an annular abscess. The patient underwent emergency mitral and aortic valve replacement. Intraoperative findings included extensive destruction of the anterior mitral leaflet and a BAV. Blood cultures grew *G. morbillorum*. The patient recovered uneventfully following 6 weeks of targeted antibiotic therapy.

**CONCLUSIONS:**

Although IE caused by *G. morbillorum* is rare, it can lead to life-threatening complications such as MVA rupture and annular abscess. Early diagnosis and prompt surgical intervention are critical to achieving favorable outcomes.

## Abbreviations

AMFC
: aorto-mitral fibrous continuity
AR
: aortic regurgitation
AVR
: aortic valve replacement
BAV
: bicuspid aortic valve
IE
: infective endocarditis
LCC
: left coronary cusp
MR
: mitral regurgitation
MVA
: mitral valve aneurysm
NCC
: non-coronary cusp
RCC
: right coronary cusp
TEE
: transesophageal echocardiography
TTE
: transthoracic echocardiography

## INTRODUCTION

MVA is a rare complication of IE, with an estimated prevalence of 0.2%–0.29% among patients undergoing TEE.^1)^ MVA can lead to leaflet fragility, perforation, and severe MR.^2)^ It often develops when infection from aortic valve IE extends to the anterior mitral leaflet.^1,3,4)^ We report a rare case of MVA rupture with an annular abscess caused by *Gemella morbillorum* IE in a patient with a BAV, successfully managed with emergency double valve replacement.

## CASE PRESENTATION

**Fig. 1:**
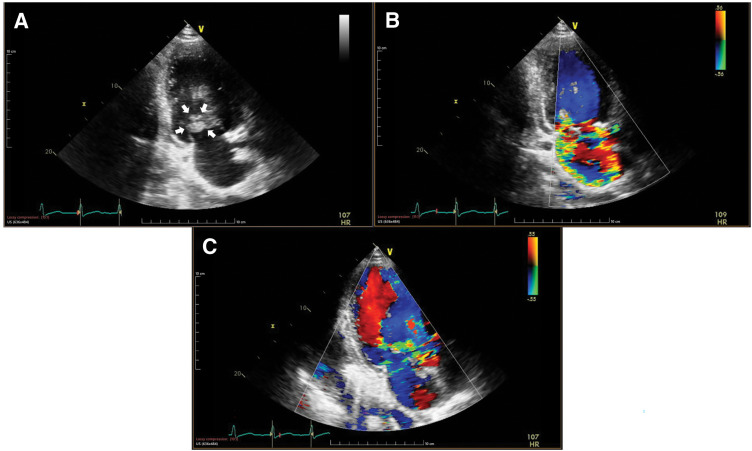
Preoperative TTE. TTE revealed marked thickening of the anterior mitral leaflet with a large mass attached to it, suggestive of either a large vegetation or aneurysmal leaflet deformation (**A**, arrows), along with severe MR (**B**) and severe AR (**C**). AR, aortic regurgitation; MR, mitral regurgitation; TTE, transthoracic echocardiography

**Fig. 2:**
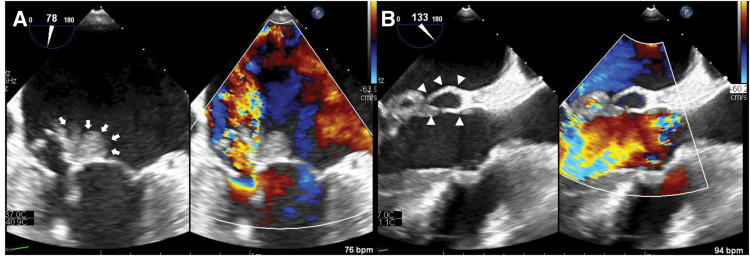
Preoperative TEE. TEE revealed an aneurysm of the anterior mitral leaflet with an intralesional perforation accompanied by a regurgitant jet (**A**, arrows). Severe AR and an echo-free space suggestive of an annular abscess beneath the NCC are also observed (**B**, arrowheads). AR, aortic regurgitation; NCC, non-coronary cusp; TEE, transesophageal echocardiography

**Fig. 3:**
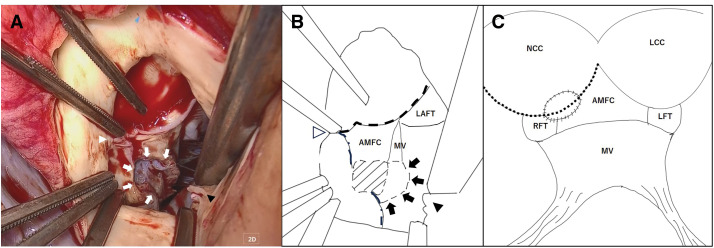
Intraoperative findings and schematic illustrations. (**A**) Intraoperative view from the aortic root. An annular abscess is observed around the aortic annulus beneath the NCC, with internal pus accumulation (white arrows). The commissures between the NCC and LCC (white arrowhead) and between the NCC and RCC (black arrowhead) are indicated. (**B**) Schematic illustration corresponding to panel A. The abscess cavity is shown (shaded area) with pus (black arrows). The commissures are marked (framed white arrowhead: L–N commissure; black arrowhead: N–R commissure). The annular margins of the LCC (dashed line) and NCC (long dashed line) are delineated. (**C**) Schematic illustration after reconstruction of the aortic annulus with an autologous pericardial patch. The abscess cavity is closed and reconstructed using a sufficiently large autologous pericardial patch that extended beyond the eroded NCC annulus and covered both the NCC annulus and the AMFC between the mitral and aortic annuli as a single continuous repair. The patch was secured circumferentially with a single continuous suture, and the suture line for aortic valve replacement is established at the level of the original aortic annulus (dotted line). AMFC, aorto-mitral fibrous continuity; LAFT, left anterior fibrous trigone; LCC, left coronary cusp; LFT, left fibrous trigone; MV, mitral valve; NCC, non-coronary cusp; RCC, right coronary cusp; RFT, right fibrous trigone

**Fig. 4:**
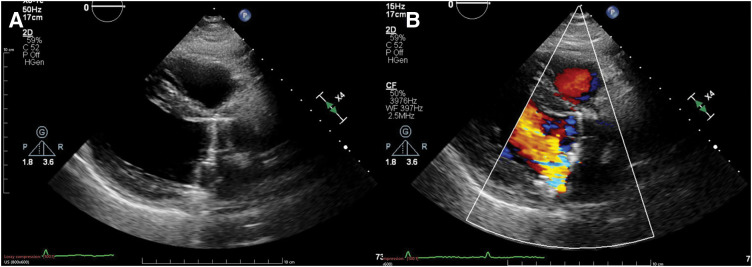
Follow-up TTE. Outpatient echocardiography reveals preserved valve function with complete closure of the abscess cavity. TTE, transthoracic echocardiography

**Fig. 5:**
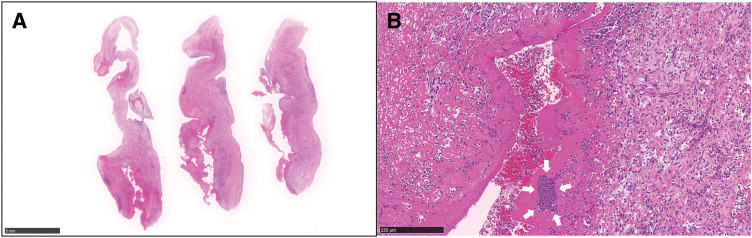
Histopathological findings of the anterior mitral leaflet (hematoxylin and eosin stain). (**A**) Low magnification (×0.52): Extensive granulation tissue with fibrin deposition and dense neutrophilic infiltration, replacing normal leaflet architecture. (**B**) High magnification (×10.2): Aggregates of bacterial colonies (white arrows) embedded within thrombotic material, consistent with active IE. IE, infective endocarditis

A 36-year-old man presented with general malaise for 1 month, followed by fever and cough for 2 weeks. He was referred to our hospital with a diagnosis of acute congestive heart failure after bilateral pleural effusion and pulmonary edema were detected at a local clinic. On admission, he was alert, with a mild fever (37.8°C), tachycardia (112 bpm), and hypoxemia (SpO^2^ 89% on room air, improving to 96% with 6 L/min oxygen). Auscultation revealed a Levine grade 5/6 systolic murmur and a diastolic murmur, with coarse crackles heard bilaterally. Chest radiography showed marked pulmonary congestion. TTE revealed a preserved left ventricular ejection fraction (70%) and marked thickening of the anterior mitral leaflet with a large mass attached to it, which was suggestive of either a large vegetation or aneurysmal leaflet deformation. Severe MR and severe AR were also noted (**Fig. 1**). An annular abscess between the NCC and LCC of the aortic valve was suspected, but could not be definitively assessed on TTE. The right ventricular systolic pressure was 58 mmHg, and the inferior vena cava diameter was 24 mm with respiratory variation. The patient was diagnosed with IE involving both the aortic and mitral valves, complicated by acute MVA and an aortic annular abscess, resulting in decompensated acute congestive heart failure. Despite oxygen therapy, dyspnea persisted, and the patient was admitted to the ICU under noninvasive positive pressure ventilation. Given the diagnosis of IE with hemodynamic compromise, emergency surgery was indicated. TEE performed after induction of anesthesia revealed an aneurysm of the anterior mitral leaflet with perforation and a regurgitant jet (**Fig. 2A**). Severe AR and an echo-free space suggestive of an annular abscess beneath the NCC were also identified (**Fig. 2B**). Surgery was performed under general anesthesia via median sternotomy. Cardiopulmonary bypass was established using aortic cannulation and bicaval venous cannulation. Upon aortotomy, a Type 1 BAV with fusion of the LCC and RCC was identified. Vegetation and an annular abscess were observed on the NCC. The lesion represented abscess cavity formation due to severe inflammatory erosion, predominantly involving the subvalvular tissue and extending from the aortic annulus to the AMFC. Although the AMFC showed localized inflammatory involvement with partial thinning, there was no full-thickness discontinuity or communication with the left atrium, and its structural continuity was preserved (**Fig. 3A** and **3B**). Via right-sided left atriotomy, the mitral valve showed a completely aneurysmal anterior leaflet with marked fragility and perforation. The anterior leaflet was resected, with half submitted for histopathology and half for culture. Vegetation on the P3 segment and ruptured chordae tendineae were also resected and examined. No definite tissue destruction of the mitral annulus due to inflammatory extension was observed. Consistent with the aortic findings, there was no apparent communication between the left atrium and the aortic lumen, and the AMFC remained preserved structural continuity. A specimen from the abscess beneath the NCC was collected for culture. After curettage and irrigation, the abscess cavity was closed and reconstructed using a sufficiently large autologous pericardial patch that extended beyond the eroded NCC annulus and covered both the NCC annulus and the AMFC between the mitral and aortic annuli. The patch was secured circumferentially with a single continuous suture, and the suture line for AVR was established at the level of the original aortic annulus (**Fig. 3C**). AVR with a 23-mm On-X mechanical valve (On-X Life Technologies, Austin, TX, USA) was performed in the supra-annular position using pledgeted non-everting mattress sutures placed from the left ventricular side to the aortic side. Mitral valve replacement was performed with a 25-mm On-X mechanical valve in the intra-annular position using pledgeted everting mattress sutures. Based on intraoperative findings, the patient, who had a congenital BAV, was found to have IE involving both the aortic and mitral valves, which had progressed to an MVA and an aortic annular abscess. Intraoperative TEE confirmed no paravalvular leakage, residual vegetation, or flow into the abscess cavity. Postoperatively, the patient was extubated on POD 2 and transferred to the general ward on POD 7. Empiric antibiotic therapy with vancomycin and meropenem was initiated, then de-escalated to vancomycin and ceftriaxone on POD 3 after Gram-positive cocci were detected. On POD 11, *G. morbillorum* was identified from the admission blood culture, and antibiotic therapy was switched to penicillin G. The regimen was continued for 6 weeks, and the patient was discharged on POD 44. Postoperative brain MRI showed no evidence of embolism or intracranial aneurysm. Outpatient follow-up demonstrated preserved valve function with complete closure of the abscess cavity (**Fig. 4**). Histopathology of the resected anterior mitral leaflet showed active IE with inflammation, necrosis, and bacterial colonies. The leaflet was almost entirely replaced by pathological tissue, with no identifiable normal architecture. No malignancy or granulomatous changes were noted (**Fig. 5**).

## DISCUSSION

Rupture of an MVA can result in acute MR, embolic events, and reinfection, leading to rapid hemodynamic deterioration.^2)^ The mechanisms of MVA formation in IE include: (1) direct extension of infection from the aortic valve through the AMFC, (2) seeding of infection onto the anterior mitral leaflet by the AR jet, and (3) contact with vegetations prolapsing into the left ventricular outflow tract (“mitral kissing vegetations”).^1,3,4)^ Non-infectious mechanisms have also been proposed, such as progressive expansion of the leaflet under elevated left ventricular pressure, resulting in a saccular aneurysm.^5)^ In our case, extensive aneurysmal change and perforation of the anterior mitral leaflet were associated with aortic valve vegetations and an annular abscess. The IE was likely related to a congenital BAV, resulting in chronic AR, which facilitated infection spread toward the mitral valve. Vegetations contacting the AMFC likely contributed to infection propagation via kissing vegetations, leading to leaflet fragility. Furthermore, chronic elevation of left ventricular pressure due to latent AR in the setting of congenital BAV may have contributed to progressive early non-infectious bulging and thinning of the anterior mitral leaflet, creating a structurally weakened substrate. Once IE developed, this compromised leaflet rapidly progressed to aneurysmal deformation and eventual perforation.

Echocardiographically, MVA appears as a saccular structure protruding into the left atrium, expanding during systole and collapsing during diastole. Differential diagnoses include severe mitral valve prolapse, large vegetations, and atrial myxoma.^6)^ TEE provides high sensitivity and specificity for detecting leaflet aneurysm, perforation, and differentiating these from other pathologies.^7–9)^

*Gemella* spp. are commensal Gram-positive cocci found in the oral cavity. Although rare, *G. morbillorum* is the most frequently reported *Gemella* spp. causing IE. The advent of molecular diagnostic techniques has improved pathogen detection in cases undiagnosed by conventional culture.^10–12)^ Despite appropriate therapy, *G. morbillorum* IE remains associated with significant morbidity and mortality, particularly with delayed diagnosis.^13,14)^ Severe cases are not uncommon; since 2000, a total of 37 reported cases of *G. morbillorum* IE with echocardiographic findings, including our own, have been documented, among which three were fatal (**Table 1**^14–48)^). BAV itself is a recognized risk factor for IE.^49)^ In a recent European cohort of 3000 IE cases, dental-origin pathogens predominated among patients with BAV, with only a single case attributed to *Gemella* spp.^50)^ Therefore, *G. morbillorum* IE complicated by MVA rupture and annular abscess represents an exceptionally rare clinical entity. Approximately one-third of reported IE cases involved multiple valves. Annular abscesses were described in four cases, whereas no cases of MVA were identified (**Table 1**^14–48)^). The patient had no recent history of dental procedures, endoscopic procedures, or clinically diagnosed gastroenteritis, and no identifiable major precipitating events. However, minor and often unrecognized occurrences—such as small oral mucosal injuries, mild gastrointestinal disturbances that typically resolve without medical attention, or minor anal fissures—could have facilitated bacterial translocation of *G. morbillorum*. Such translocation, in the setting of congenital BAV, was considered the most plausible mechanism underlying the development of IE. Successful management was achieved through emergency valve replacement and targeted antibiotic therapy.

**Table 1:** Reported cases of *Gemella morbillorum* infective endocarditis with echocardiographic findings since 2000

Number	Author	Year	Age	Sex	IE region	MVA	Annular abscess	surgical procedure	Outcome
No. 1	Farmaki et al.^48)^	2000	9	F	M	–	–	none	Alive
No. 2	Akiyama et al.^47)^	2001	55	M	A	–	–	AVR	Alive
No. 3	Al Soub et al.^46)^	2003	41	F	M	–	–	MVP	No explained
No. 4	Woo et al.^45)^	2003	66	M	A, M	–	–	none	Alive
No. 5	Zakir et al.^44)^	2004	44	M	M	–	+	none	Alive
No. 6	Kofteridis et al.^43)^	2006	46	M	M	–	–	none	Alive
No. 7	Kofteridis et al.^43)^	2006	53	M	M	–	–	none	Alive
No. 8	Murai et al.^42)^	2006	53	M	M	–	–	MVR	Alive
No. 9	Zheng et al.^41)^	2008	67	M	A, M	–	–	none	Died
No. 10	Seeburger et al.^40)^	2009	76	M	A, M, T, P	–	–	Quadruple valve replacement	Alive
No. 11	Al Chekakie et al.^39)^	2009	44	M	A	–	+	AVR	Died
No. 12	Hull^38)^	2010	87	M	M	–	–	none	Alive
No. 13	Carano et al.^37)^	2010	18	F	M	–	–	MVR	Alive
No. 14	Taimur et al.^36)^	2010	31	F	A	–	–	none	Alive
No. 15	Godinho et al.^35)^	2013	72	M	A, M, T	–	–	DVR + TAP	Alive
No. 16	Shahani^34)^	2014	73	M	A	–	+	AVR	Alive
No. 17	Agrawal et al.^33)^	2014	–	–	P	–	–	PVR + ASD closure	Alive
No. 18	Ural et al.^14)^	2014	67	M	A	–	–	none	Alive
No. 19	Kolhari et al.^32)^	2014	34	F	M	–	–	MVR	Alive
No. 20	Constantinos et al.^31)^	2015	80	F	T	–	–	none	Alive
No. 21	Rosa et al.^30)^	2015	72	M	M	–	–	MVR	Died
No. 22	Li et al.^29)^	2017	28	M	P	–	–	PVR +ASD closure + VSD closure	Alive
No. 23	Kumar et al.^28)^	2017	12	F	M	–	–	none	Alive
No. 24	Shinha et al.^27)^	2017	37	M	A	–	–	No explained	No explained
No. 25	Rehman et al.^26)^	2019	33	M	A, P	–	–	AVR + PVR + VSD closure	No explained
No. 26	Lee et al.^25)^	2019	33	M	A, P	–	+	AVR + PVR + VSD closure	Alive
No. 27	Dogan et al.^24)^	2020	37	M	A, M	–	–	DVR	Alive
No. 28	Kobayashi et al.^23)^	2021	54	M	M	–	–	MVR	Alive
No. 29	Spaeth et al.^22)^	2020	23	M	A, M	–	–	DVR	Alive
No. 30	Desai et al.^21)^	2021	72	M	A, M, P	–	–	DVR	Alive
No. 31	Tanveer et al.^20)^	2021	48	M	M	–	–	MVR	Alive
No. 32	Patel et al.^19)^	2021	56	F	–	–	–	none	Alive
No. 33	Liu et al.^18)^	2022	40s	F	M	–	–	none	Alive
No. 34	Lim et al.^17)^	2023	40	M	A, M	–	–	DVR + TAP	Alive
No. 35	Cao et al.^16)^	2023	37	M	A	–	–	none	Alive
No. 36	Panama et al.^15)^	2024	31	M	A, M	–	–	DVR	Alive
No. 37	Present case	2025	36	M	A, M	+	+	DVR with reconstruction of aortic annulus	Alive

A, aortic valve; ASD, atrial septal defect; AVR, aortic valve replacement; DVR, double valve (aorttic and mitral) replacement; IE, infective endocardaitis; IE region, infective endocarditis region; M, mitral valve; MVA, mitral valve aneurysm; MVP, mitral valve plasty; MVR, mitral valve replacement; P, pulmonary valve; PVR, pulmonary valve replacement; T, tricupid valve; TAP, tricuspid annuloplasty; VSD, ventrial septal defect

No established treatment guidelines exist for MVA. Conservative observation may be considered for uncomplicated cases.^1)^ However, aneurysm size does not correlate with rupture risk, and early surgery is recommended by some authors to prevent serious complications.^7,51)^ In particular, Nishino et al. reported a case of MVA perforation occurring 3 years after conservative treatment of a deep abscess,^52)^ indicating that even small or asymptomatic MVAs may progress and warrant surgical intervention. Symptomatic MVAs, particularly those causing heart failure or embolic events, often require urgent or emergency surgery.^5,53)^ Our patient required emergency surgery for heart failure with severe respiratory failure. Owing to extensive infection and tissue destruction, both aortic and mitral valves were replaced. While repair may be feasible in limited cases, valve replacement is indicated in extensive disease, as in our case.^51,54)^

## CONCLUSIONS

Emergency double valve replacement was successfully performed for MVA rupture and annular abscess associated with *G. morbillorum* IE, leading to a favorable outcome. Although rare, MVA can be life-threatening; early diagnosis and timely surgical intervention are essential.
